# When Being Overweight Masks the Diagnosis: Identifying Familial Hypercholesterolemia in Pediatric Patients

**DOI:** 10.7759/cureus.85836

**Published:** 2025-06-12

**Authors:** Mariam Oniani, Nino Kheladze, Salome Kaldani

**Affiliations:** 1 Pediatric Endocrinology, M. Iashvili Children’s Central Hospital, Tbilisi, GEO; 2 Pediatric Endocrinology and Diabetes Center, M. Iashvili Children’s Central Hospital, Tbilisi, GEO; 3 Endocrinology, Tbilisi State Medical University, American MD Program, Tbilisi, GEO

**Keywords:** cascade screening, dyslipidemia, genetic familial hypercholesterolemia, genetic screening, obesity, pediatric metabolic diseases, statin therapy

## Abstract

Familial hypercholesterolemia (FH) is a genetic disorder leading to elevated low-density lipoprotein cholesterol (LDL-C) from early life, significantly increasing the risk of premature atherosclerotic cardiovascular disease. Despite its prevalence, FH remains underdiagnosed, particularly in pediatric populations where obesity may obscure clinical suspicion. Here, we present two Georgian siblings diagnosed with heterozygous familial hypercholesterolemia (HeFH). The index patient, a nine-year-old boy, presented with overweight and elevated LDL-C that persisted despite weight loss. Evaluation of his brother, with a normal body mass index, and family members revealed similar lipid profiles, prompting the diagnosis of FH. Both siblings were started on atorvastatin 10 mg daily, resulting in significant LDL-C reduction without adverse effects. This case highlights the importance of distinguishing primary from secondary causes of dyslipidemia in children. The cumulative cholesterol burden from early life necessitates timely diagnosis and intervention. Obesity may mask FH, contributing to delays in recognition and management. Early clinical suspicion, comprehensive family screening, and timely initiation of statin therapy are essential for effective management of pediatric HeFH and the prevention of long-term cardiovascular complications.

## Introduction

Dyslipidemia refers to abnormalities in one or more lipid parameters and may arise from dietary habits, secondary conditions such as type 2 diabetes mellitus, hypothyroidism, or obesity, as well as from genetic disorders [1]. According to data from the National Health and Nutrition Examination Survey, lipid disorders affect 20% of adolescents aged 12 to 19 years. This prevalence is even greater among children with obesity, reaching 42% [2].

Familial hypercholesterolemia (FH) is a common autosomal dominant disorder of lipoprotein metabolism, causing elevation of low-density lipoprotein cholesterol (LDL-C) from the beginning of a patient’s life. If left untreated, FH can lead to lifelong dyslipidemia and increase the risk of premature atherosclerotic cardiovascular disease (ASCVD). Although the prevalence of heterozygous familial hypercholesterolemia (HeFH) is approximately 1:220, it is still underdiagnosed and undertreated [3]. Untreated men and women face a 30-50% likelihood of experiencing either fatal or nonfatal cardiac events by the ages of 50 and 60 years, respectively [4].

The genes primarily affected in FH code for the LDL receptor, B apolipoprotein, or proprotein convertase subtilisin/kexin 9 (PCSK9), resulting in increased levels of LDL-C since birth [5]. While cardiovascular diseases are not typically seen in children, studies have shown that the process of atherosclerosis begins during childhood, further supporting the need for early identification and treatment  [6].

HeFH can be diagnosed using clinical criteria and through molecular genetic testing. It should be noted that normal pediatric lipid profile values are lower than those for adults: optimal and borderline high range of LDL-C <110 mg/dL and 110-129 mg/dL, respectively [7]. The diagnosis of FH should be considered when LDL-C level is persistently elevated with at least one or more of the following: family history of hypercholesterolemia, history of premature ASCVD in the patient or family members, or physical examination typical of hypercholesterolemia (such as xanthomas, xanthelasmas) [8].

Management of FH consists of several aspects, including the implementation of proper education, encouragement of physical activity, dietary interventions, and pharmacological treatment [3]. The objective of treatment is to lower LDL-C to a minimum level of less than 130 mg/dL (3.4 mmol/L), with an ideal target of below 100 mg/dL (2.6 mmol/L) [8]. Although lifestyle changes are the initial strategy in lowering LDL-C levels, they are rarely sufficient [9]. Pharmacological treatment needs to be considered after three to six months of adopting the appropriate lifestyle. Statins are the first drug of choice in children older than eight years [4,9,10].

When dyslipidemia is present, it is crucial to exclude the secondary causes such as obesity, hypothyroidism, and chronic kidney and liver failure. As FH is relatively uncommon in routine pediatric practice, its coexistence with secondary causes of dyslipidemia, such as obesity or endocrine disorders, can obscure clinical suspicion and delay diagnosis. This case report aims to highlight the significance of early diagnosis and treatment of HeFH, particularly in contexts where the prevalence of obesity may obscure the recognition of FH, making timely intervention critical for effective management.

## Case presentation

A nine-year-old Georgian boy from Tbilisi was referred to the pediatric clinic due to being overweight. According to his parents, the boy was physically active, attending swimming classes three times a week, but consumed a large amount of sweets and sweetened beverages daily. Physical examination revealed a healthy boy at Tanner stage 1 with no signs of acanthosis nigricans, xanthomas, or hepatosplenomegaly. His body mass index (BMI) was at the 85th percentile for his age and sex. The family history was negative for any chronic or genetic disorders. Initial laboratory testing showed an elevated LDL-C level of 217.13 mg/dL (Table 1), despite normal thyroid function and liver enzymes (Table 2). Based on the patient’s history and laboratory analysis, his excess weight was attributed to lifestyle factors, and he was referred to a dietitian for proper counseling on nutrition and physical activity.

**Table 1:** Lipid profile trends in two siblings diagnosed with heterozygous familial hypercholesterolemia. This table presents the longitudinal lipid profile data for two Georgian siblings diagnosed with heterozygous familial hypercholesterolemia. Lipid parameters, including total cholesterol, LDL-C, HDL-C, triglycerides, and VLDL-C, are recorded across multiple time points spanning from March 1, 2024, to March 22, 2025. Both siblings underwent dietary modifications and statin therapy during this period, with notable improvements in LDL-C levels. Each value represents the laboratory-measured concentration (mg/dL) during the specified dates. LDL-C: low-density lipoprotein cholesterol; HDL-C: high-density lipoprotein cholesterol; VLDL-C: very low-density lipoprotein cholesterol

Laboratory value	Patient 1						Patient 2			
Date	March 1, 2024	June 20, 2024	July 4, 2024	September 5, 2024	January 25, 2025	March 22, 2025	July 4, 2024	September 5, 2024	January 25, 2025	March 22, 2025
Total cholesterol (mg/dL)	261.7	283.5	205.3	287.3	259.3	142.0	247.2	258.2	267.0	176.0
LDL-C (mg/dL)	217.13	215.9	152.54	209.74	183.7	67.0	183.5	191.7	193.4	111.0
HDL-C (mg/dL)	46.5	50.1	42.1	50.6	57.6	55.6	50.1	48.3	53.4	53.5
Triglycerides (mg/dL)	98.7	85.7	103.9	137.7	76.2	120.0	91.4	85.5	74.7	83.0
VLDL-C (mg/dL)	19.7	17.1	20.8	27.5	15.2	24.0	18.3	17.1	14.9	16.0

**Table 2:** Liver function tests, thyroid function, and creatine linase levels in siblings with familial hypercholesterolemia. This table details the liver function tests (ALT, AST), TSH levels, and creatine kinase (CK) values for both siblings diagnosed with familial hypercholesterolemia. Measurements were taken at several clinical follow-up points between March 1, 2024, and April 28, 2025. These parameters were monitored to evaluate the safety and tolerability of statin therapy, ensuring no adverse hepatic or muscular effects during treatment. LDL-C: low-density lipoprotein cholesterol; HDL-C: high-density lipoprotein cholesterol; VLDL-C: very low-density lipoprotein cholesterol; AST: aspartate transaminase; ALT: alanine transaminase; CK: creatine kinase; TSH: thyroid-stimulating hormone

Laboratory value	Patient 1				Patient 2					
Date	January 25, 2025	February 4, 2025	March 22, 2025	April 28, 2025	March 1, 2024	June 20, 2024	January 25, 2025	February 4, 2025	March 22, 2025	April 28, 2025
ALT	-	9.0 U/L	13.0 U/L	-	-	-	-	8.0 U/L	10.0 U/L	-
AST	-	21.0 U/L	25.0 U/L	-	-	-	-	21.0 U/L	24.0 U/L	-
CK	-	-		42 U/L	-	-	-	-	-	42 U/L
TSH	1.287 µIU/mL	-	1.678 µIU/mL		1.311 µIU/mL	1.843 µIU/mL	0.826 µIU/mL	-	1.126 µIU/mL	-

Despite losing 4 kg over three months, his lipid levels remained unchanged (Table 1). After an additional weight loss of 3 kg over the next three months, lipid levels were rechecked but persisted at high levels (Table 1). This prompted an investigation into other causes of pediatric dyslipidemia. The patient’s seven-year-old brother, who had a normal BMI for his age and sex, was assessed and also found to have dyslipidemia. Further examination of family members revealed that both the father and grandmother had dyslipidemia. A clinical diagnosis of HeFH was made. The family was educated regarding the chronicity of the HeFH and the treatment options. Initiation of statin therapy was decided. Liver function tests and creatine kinase were evaluated before starting treatment.

After three months of taking 10 mg of statin daily, the dyslipidemia was resolved (Table 1). The family was counseled again on the importance of treatment adherence, proper lifestyle choices, nutrition, and routine follow-ups.

## Discussion

Overweight children frequently present with dyslipidemia, which typically improves with lifestyle modifications, including dietary changes and increased physical activity [11]. However, when elevated lipid levels persist despite weight loss, clinicians must evaluate for primary or genetic causes of dyslipidemia.

FH is an autosomal dominant disorder characterized by elevated LDL-C from birth. Due to its silent nature and the absence of universal lipid screening protocols in many regions, most cases remain undiagnosed until adulthood. Nevertheless, atherosclerotic changes begin in childhood, underscoring the importance of early recognition and management. The concept of “cumulative cholesterol burden” refers to the lifetime exposure of arterial walls to elevated LDL-C and is a strong predictor of ASCVD. A recent study found that an increase of 75 mmol in cumulative LDL-C exposure can double coronary plaque volume [12].

This case involved a nine-year-old boy referred for being overweight. Although lifestyle changes led to measurable weight loss, his LDL-C levels remained elevated, prompting further evaluation. His seven-year-old brother, who had a normal BMI, also showed dyslipidemia. Family screening revealed elevated cholesterol levels in the father and grandmother, leading to a clinical diagnosis of HeFH based on the American Heart Association (AHA) criteria. Both siblings were started on atorvastatin 10 mg daily, which led to a significant reduction in LDL-C without adverse effects.

According to current guidelines, pharmacologic therapy should be initiated in children older than eight years if LDL-C remains elevated after three to six months of lifestyle modification [4,9,10]. Statins are the first-line agents and are generally well tolerated in pediatric populations. Both siblings in this report achieved LDL-C improvement without signs of statin-induced myopathy (Table 1).

Cardiovascular disease remains the leading cause of death worldwide [13]. Early detection and management of HeFH in childhood can significantly reduce future ASCVD risk. This case emphasizes the importance of maintaining clinical suspicion for genetic dyslipidemias, even when secondary factors such as obesity are present, as they can obscure the diagnosis. Furthermore, it highlights the significance of cascade screening for the early detection of affected family members. Implementing universal pediatric lipid screening during childhood and adolescence, specifically between the ages of 9 and 11 years and again between 17 and 21 years, serves as a crucial strategy to prevent missed diagnoses and reduce long-term cardiovascular risks [8,14].

## Conclusions

FH causes lifelong LDL-C elevation and significantly increases the risk of premature ASCVD, yet it often goes unrecognized in children, particularly when obesity masks clinical suspicion. As cardiovascular disease is the leading cause of death globally, early identification and timely lipid-lowering therapy are crucial to reduce long-term risk. In the absence of molecular confirmation, pharmacologic intervention should be initiated after six months of lifestyle modifications if LDL-C targets are not met. Management strategies should be tailored to the patient’s age, pubertal status, and family history, with close monitoring for therapeutic efficacy and safety. Cascade screening and genetic counseling are critical components for identifying at-risk family members and preventing missed diagnoses. This case highlights the importance of clinical suspicion in overweight pediatric patients with persistent dyslipidemia and supports universal lipid screening during childhood and adolescence as an effective strategy to reduce cardiovascular risk and improve long-term outcomes.
